# Fitting Transporter Activities to Cellular Drug Concentrations and Fluxes: Why the Bumblebee Can Fly

**DOI:** 10.1016/j.tips.2015.07.006

**Published:** 2015-11

**Authors:** Pedro Mendes, Stephen G. Oliver, Douglas B. Kell

**Affiliations:** 1 School of Computer Science; 2 Manchester Institute of Biotechnology, The University of Manchester, 131 Princess St, Manchester M1 7DN, UK; 3 Centre for Synthetic Biology of Fine and Speciality Chemicals (SYNBIOCHEM), The University of Manchester, 131, Princess St, Manchester M1 7DN, United Kingdom; 4 Center for Quantitative Medicine, University of Connecticut, UConn Health, 263 Farmington Avenue, Farmington, CT 06030-6033, USA; 5 Cambridge Systems Biology Centre; 6 Dept of Biochemistry, University of Cambridge, Sanger Building, 80 Tennis Court Road, Cambridge CB2 1GA, UK; 7 School of Chemistry, The University of Manchester, Manchester M13 9PL, United Kingdom

## Abstract

A recent paper in this journal argued that reported expression levels, k_cat_ and K_m_ for drug transporters could be used to estimate the likelihood that drug fluxes through Caco-2 cells could be accounted for solely by protein transporters. It was in fact concluded that if five such transporters contributed ‘randomly’ they could account for the flux of the most permeable drug tested (verapamil) 35% of the time. However, the values of permeability cited for verapamil were unusually high; this and other drugs have much lower permeabilities. Even for the claimed permeabilities, we found that a single ‘random’ transporter could account for the flux 42% of the time, and that two transporters can achieve 10 · 10^−6^ cm·s^−1^ 90% of the time. Parameter optimisation methods show that even a single transporter can account for Caco-2 drug uptake of the most permeable drug. Overall, the proposal that ‘phospholipid bilayer diffusion (of drugs) is negligible’ is not disproved by the calculations of ‘likely’ transporter-based fluxes.

## Trends

There has been recent debate as to the relative extents to which cellular transmembrane drug transports occur through any phospholipid bilayer region or is transporter-mediated only.

Much recent evidence suggests (perhaps surprisingly) that phospholipid bilayer diffusion is negligible.

A recent article in this journal suggested that the expression profile and kinetics of known transporters might not be adequate to explain the most active drug fluxes (of verapamil and propranolol) in Caco-2 cells via transporters only.

We show with our own simulations that this is not in fact the case, especially when evolutionary selection is taken into account, and that the Haldane relation accounts straightforwardly for directional differences, even for equilibrative transporters.

Typical protein transporters alone can easily account for measured drug fluxes in Caco-2 cells.

## Pre-eminence of Transporter-Mediated Drug Uptake

For cases in which a drug must interact with one or more intracellular targets, and for all oral drugs, it is necessary for drugs to cross at least one biomembrane. There is an increasing recognition that to cross intact biological membranes drugs must or do hitchhike on transporters that are normally involved with intermediary metabolism (e.g. 1, 2, 3, 4, 5, 6, 7, 8, 9, 10, 11, 12). It is therefore of interest to understand how the use of specific influx and efflux transporters translates into particular transmembrane fluxes and intracellular concentrations (and hence the biological effects of drugs and other solutes). A recent example [13] brings the issue into sharp focus, where removing (genetically) just a single transporter decreased the toxicity (and presumably accumulation) of the drug YM155 (sepantronium bromide) by several hundred-fold. The implication of such data is that any ‘background’ rate involving phospholipid bilayer diffusion must be rather less than 1%, or (as we have put it elsewhere 9, 10) ‘phospholipid bilayer diffusion is negligible’. Another recent example (see Figure 2 in ref [14]) shows that metformin uptake can be accounted for entirely by four transporters. Indeed, this essential lack of permeability in the absence of suitable transporters readily accounts for the failure of drugs to penetrate to the sites where they are required. Anti-tuberculosis drugs provide another important and (for patients) damaging example 15, 16.

**Fig. 1:**
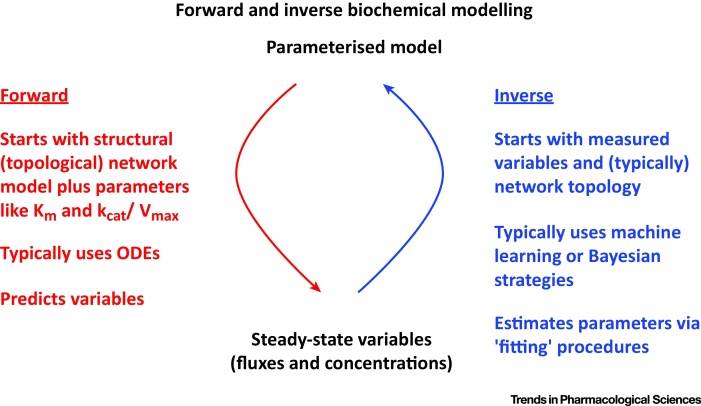
Relationships between Forward and Inverse Modelling. In forward (ODE-based) modelling, parameters such as the network topology, enzyme concentrations, k_cat_ and K_m_ are the inputs and variables such as fluxes and concentrations are the output 18, 19. In inverse modelling the inputs are the variables such as fluxes and concentrations, and one must determine or estimate the parameters (and maybe even the network topology) that permits such variables to occur.

The nonlinear nature of many biochemical kinetics, and the complex behaviour of even simple biochemical pathways, means that it is hard to ‘guess’ what might happen without seeking to model it first (e.g. 17, 18, 19). Thus, a recent article in this journal [20] (and its subsequent supplementary iformation [21]) sought to carry out just such a modelling study, based on a series of stated assumptions. The authors [20] also drew a major conclusion that (we consider) was at some variance with the data presented. The two main purposes of the present paper are (i) to go through their data and main argument, and, (ii) because natural evolution has at least one selection step, to study what happens when instead of making assumptions solely about forward modelling, one simply fits the observables to appropriate models and their parameters (Figure 1).

## A Note on the Word ‘Passive’ and Why One Should Use More Explicit Alternatives

**Fig. 2:**
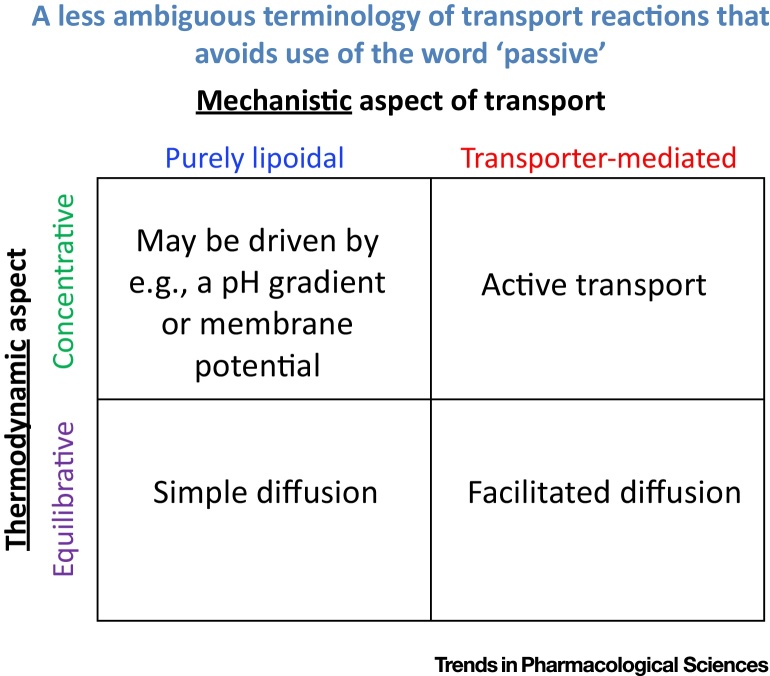
Two Orthogonal Aspects of Cellular Uptake in which the Word ‘Passive’ Is Sometimes (and Unhelpfully) Used to Describe (and, in the Worst Cases, Conflate) Two Completely Different Concepts. The first is a thermodynamic usage meaning ‘equilibrative’, for which the antonym is ‘active’ or better ‘concentrative’. The second usage is intended to be a mechanistic usage, and is sometimes taken to mean ‘via bilayer lipoidal bilayer diffusion’, in which case it is best to state this. Carrier-mediated but equilibrative diffusion is historically referred to as ‘facilitated diffusion’. Needless to say, showing that transport is equilibrative (or ‘passive’) does not explain whether its uptake is transporter-mediated or otherwise. To avoid any such ambiguity, we suggest strongly that all workers simply avoid the word passive entirely, and replace it with words that describe precisely and explicitly which of the two meanings (thermodynamic vs mechanistic) is intended.

Despite our clear previous explanation of this term [9], Matsson and colleagues [20] (and many other workers) continue to use the word ‘passive’ to mean two entirely different things (Figure 2). The first usage involves a thermodynamic statement only, and is best referred to as ‘equilibrative’ (‘passive’ transport is thermodynamically equilibrative; the ‘active’ version requires an input of free energy and is then concentrative). We would stress that, as such, the word ‘passive’ has nothing of itself to say about a mechanism of how a drug crosses a membrane. However, ‘passive’ transport is also far too often taken to mean ‘transport via bilayer lipoidal’ diffusion, a perfectly acceptable intent provided this is made explicit, but one that is then best served by calling it ‘bilayer lipoidal diffusion’ directly. Carrier-mediated diffusion may be active or passive in the thermodynamic sense (and, for those purposes, is best referred to as either concentrative or equilibrative). A very well-established term for the latter (carrier-mediated equilibrative transport) is ‘facilitated diffusion’, while the term ‘active transport’ is perfectly adequate for concentrative transporter-mediated solute influx (or efflux). All of this therefore entirely avoids the ambiguity common with the use of the term ‘passive’. We reiterate strongly that much trouble would be avoided if the word ‘passive’ were dropped completely from all debates about transmembrane drug uptake mechanisms. Conflating the two by showing its truth for one meaning (thermodynamic) but then claiming that this thereby shows the other meaning of bilayer lipoidal is at best unscientific. (Zheng and colleagues [22] illustrate this with an example in which bilayer transport was not even measured directly as a dependent variable, and for a drug whose uptake is stereoselective and hence necessarily transporter-mediated.)

## Fluxes across Caco-2 Cell Membranes Explicable Via Transporter Reactions

Matsson and colleagues [20] proposed, as a model, the well-known Caco-2 cell system, and sought to estimate how ‘likely’ it was, given the known expression profiles and k_cat_ values of a subset of transporters, whether or not they could reasonably be expected to account for the fluxes observed in the case of two drugs (propranolol and verapamil) with unusually high permeabilities. At first glance, this is an interesting idea. Note that Caco-2 cells are thought (from transcriptomics or proteomics measurements) to express several hundred (e.g. 23, 24, 25) of the ca 450 catalogued SLC transporters, although (i) there is considerable variation in this between laboratories [26], (ii) it is not known how reliable the expression profiling data are [26], and (iii) it is recognised that ‘unknown’ transporters might be present. Thus, some of the authors of [20] already published that there is an enormous expression level of an ‘HPT1’ human peptide transporter 26, 27 (indeed it is the highest expressed transporter in Caco-2 cells in each of the 10 laboratories participating in [26]), but such a transporter seems to make no appearance at all in [20]. Thus, in the absence of any knowledge, nor of the inclusion of such highly expressed transporters, these estimates are always likely to be underestimates. We entirely appreciate the complexities of biological systems, and hence, the difficulty of reproducing the behaviour of even the well-established Caco-2 system. However, to give an indication of the variance observable within and between laboratories, Box 1 shows some of the data from precisely such a comparison [26]. Obviously the variance between laboratories for the three drugs atenolol, metoprolol and talinolol is at least an order of magnitude (sometimes more), with their median values for A → B being ca 0.5, 45 and 1.34 · 10^−6^ cm·s^−1^.

Regarding the choice of drugs, Mattson and colleagues [20] state “Classical examples include propranolol and verapamil. These have permeability coefficients across Caco-2 intestinal epithelial cell monolayers (the most commonly used cellular barrier for permeability studies) in the range 200–1000 · 10^−6^ cm·s^−1^ 28, 29.” Actually the rate published for R- or S-verapamil in [28] was ∼100 · 10^−6^ cm·s^−1^, and even decreased as concentrations exceeded 100 μM, presumably because of substrate inhibition, with a similar value in [29]. Some of the authors of Matsson *et al.* [20] in their reference 19 [30] published a value of 155 · 10^−6^ cm·s^−1^, that for propranolol in Artursson and Karlsson [31] was 41.9 · 10^−6^ cm·s^−1^, in Camenisch *et al*. [32] 41.7 · 10^−6^ cm·s^−1^, van Breemen and Li [33] gave 50 · 10^−6^ cm·s^−1^, while that for propranolol in Figure 3 of [29] was ∼700 · 10^−6^ cm·s^−1^, but no matter. Corti and colleagues [34] (their Table 2) give 41.9, 10^6^ cm·s^−1^ for propranolol and 15.8 · 10^−6^ cm·s^−1^ for verapamil. This said, the ‘observable’ rates stated in Figure 3A(i) of [20] as 1310 · 10^−6^ cm^−1^ for verapamil and 230.10^−6^ cm^−1^ for propranolol come from Table 3 of a paper by Avdeef [29] (P. Matsson, personal communication), and are obviously at some variance with these other numbers. (They are based on a very rapid stirring – 700 rpm – that does not occur adjacent to natural epithelia.) Anyway, although these high values are close to being complete outliers (Table 1), we shall take the larger numbers as given, and the question arises as to whether typical fluxes of individual carriers can come close to being able to achieve these overall values of *P*_app_.

The authors [20] (and most of the data have subsequently been made available as Supplementary Information [21]), took random samples of individual transporters whose k_cat_ values (for just 18 transporters using unstated substrates), K_m_ and expression levels were drawn from a random distribution of a known subset. Note the wide variation for each one – in Figure 2B of [20] the k_cat_ value for VMAT2 varied 200-fold). They found [20] that that the observed rates for verapamil and propranolol at 50 μM were reached in 7% and 18% of cases, and that if it is was assumed that five transporters might be involved equally then this would be found for 35% of cases for verapamil (and presumably a significantly greater percentage for propranolol, though that was not stated). Presumably these drugs were chosen because of their high fluxes, albeit that their uptake shows enantioselectivity (e.g. 35, 36) and thus must be transporter-mediated, so this is very far from making this an ‘unlikely’ event. Thus, even though we consider this to be entirely the wrong strategy, this seems to us to be a rather positive endorsement of the fact that most flux is perfectly capable of going via transporters even for drugs that were apparently chosen to have the highest total rates. Matsson *et al*. [20] also comment that “marketed drugs target between one and eight distinct proteins (5th to 95th percentile range [37])”. Actually, on average each marketed drug has six known targets [38], so we may assume this is something of an underestimate. In the case of verapamil, it is transported by multiple isoforms of SLC22 39, 40, 41 among others yet uncharacterised 42, 43, as is propranolol 44, 45, so the calculations presented by Matsson and colleagues are necessarily likely to underestimate the transporter-mediated fluxes. As we have said before 6, 9, absence of evidence is not evidence of absence. It is also worth commenting that, in the absence of other knowledge, the absolute transcript level alone can be an adequate surrogate for predicting fluxes in genome-wide studies [46].

**Figure 3:**
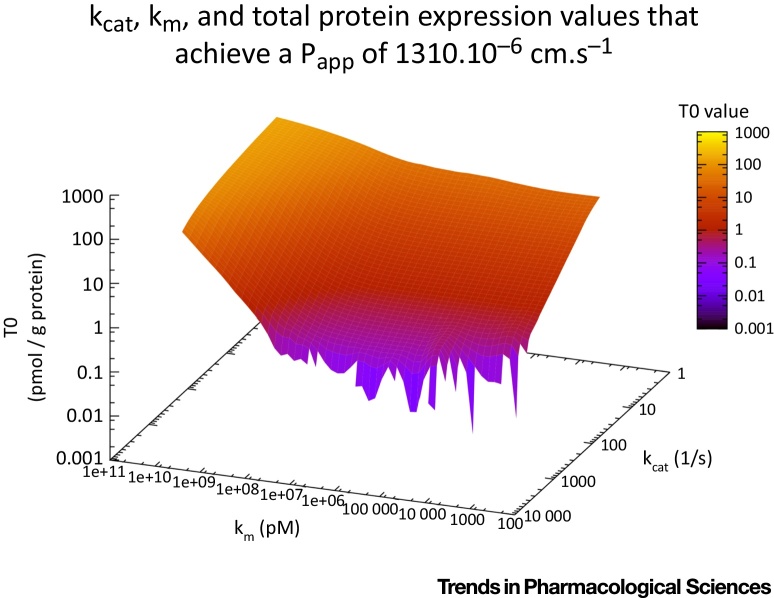
Variation of Parameters Necessary to Achieve a Flux of 1310 · 10^−6^ cm·s^−1^ in an *In Silico* Caco-2 Transport System with a Single Transporter, Coloured by the Value of the Transporter Expression. The equation and its units are given in Box 2.

However, natural (Darwinian) evolution has a selection step in it, and it is precisely this that accounts for the fact that complex organisms evolve, however ‘unlikely’ or ‘implausible’ that may be 47, 48, 49. Thus, from our perspective, the correct strategy is to start with the data and find the parameter values that can fit it for one or more transporters, and how often such a fit can be obtained 17, 50. This was performed 1000 times, and on each occasion, with just a single transporter, we could, within the bounds of the parameters given by Matsson and colleagues, achieve a flux of 1310 · 10^−6^ cm·s^−1^ on every single occasion. We therefore did not repeat the analysis with more than one transporter. The data are given in Figure 3. Two features are of note. First, and fairly obviously, is the fact that a given V_max_ can be obtained from varying the coupled values of k_cat_ and transporter concentration. Secondly, although they represent different aspects of enzyme action [51], the values for V_max_ and K_m_ are not actually completely independent of each other under selection. This is in fact related to the Haldane relationship discussed below.

## Permeabilities of Other Drugs

**Figure 4:**
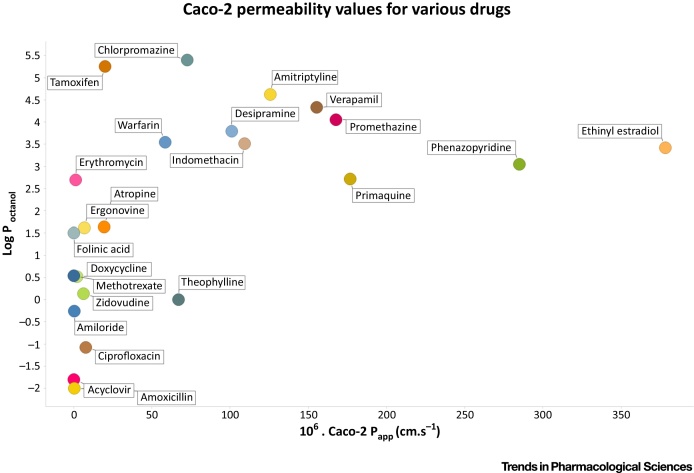
Some Values of Caco-2 Permeability of Various Drugs and Their Relative Independence from Log P. Data are replotted from Table 1 of [30].

A table of various substances’ permeability coefficients in Caco-2 cells is given in Table 1 of [30] (and stated to have been redrawn in Figure 2A of [52], though the former has 23 and the latter 31 data points). (Note that Bergström and colleagues [30] also avoided unstirred layer effects, albeit that they anyway have equal (ir)relevance to measurements of fluxes and the transporter kinetics with which they are supposed to be comparing.) We have plotted out those data (Figure 4), from which at least three conclusions are evident: (i) the *P*_app_ for very few of the compounds exceeds even 100 · 10^−6^ cm·s^−1^, and of the only two that exceed 200 · 10^−6^ cm·s^−1^, one (ethinyl estradiol) is a sterol that is heavily metabolised to its sulphate and is transported by the sterol transporter SLC51 53, 54) and (as the sulphate) by a series of anion transporters 55, 56, 57, while the other (phenazopyridine) is a rarely used local anaesthetic (and adenine analogue) that, in fact, is seen as poorly transported/metabolised (class IV) in the BDDCS system [58]; (ii) there is no discernibly linear relationship between permeability and the log of the octanol:water partition coefficient (see also 1, 9) (that we have purposely plotted on the ordinate to highlight the fact that it is not an independent variable), (iii) as previously pointed out 4, 6, 9 almost all of them do have known transporters. While the contributions of paracellular and efflux transporters is not known (and verapamil is a well known P-gp inhibitor, e.g. [59]), similar conclusions on the normally rather lower values for Caco-2 permeability may be drawn from the compilations of Artursson & Karlsson [31] (20 drugs, highest permeability 54.5 · 10^−6^ cm·s^−1^), Corti *et al*. [34] (21 drugs, highest permeability 83 · 10^−6^ cm·s^−1^), Yee [60] (∼26 drugs, highest permeability 71 · 10^−6^ cm·s^−1^), Camenisch *et al*. [32] (∼25 drugs, highest permeability 61.7 · 10^−6^ cm·s^−1^), Pade & Stavchansky [61] (9 drugs, highest permeability 45.5 · 10^−6^ cm·s^−1^), Yazdanian *et al*. [62] (51 drugs, highest permeability 36.6 · 10^−6^ cm·s^−1^), Hou *et al*. [63] (77 drugs, highest permeability 52.5 · 10^−6^ cm·s^−1^), Uchida *et al*. [64] (8 drugs, highest permeability 55.3 · 10^−6^ cm·s^−1^), and Lozoya-Agullo *et al*. (2015) [65] (15 drugs, highest permeability 41.8 · 10^−6^ cm·s^−1^). The median permeability of the drugs listed in the cited references is less than 20 · 10^−6^ cm·s^−1^, which is considerably lower than the kinds of numbers given above and highlighted in [20].

**Figure 5:**
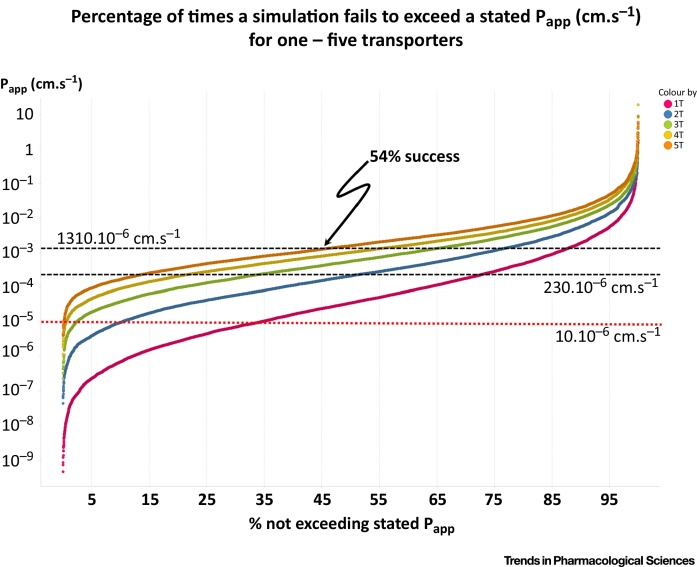
Rank Order of *P*_app_ Obtained when Parameters were Varied for 1,2,3,4 and 5 Transporters, with K_m_ = 50 μM, as in Fig. 3. A *P*_app_ of 1310 · 10^−6^ cm·s^−1^ is achieved in 12% of cases for 1 transporter and 54% of cases for 5 transporters, with correspondingly more frequent successes when *P*_app_ is lower.

As described in Box 2 and the supplementary information, we have also used COPASI to model this system using 10,000 values of K_m_, k_cat_ and protein expression drawn from the best-fit log-normal distribution given in the supplementary data [21] of [20]. A number of points follow from this Figure: (i) there is a tendency for a particular transporter to dominate, i.e. there is a law of diminishing returns, (ii) in our hands, we could achieve the ‘target’ flux of 1310 · 10^−6^ cm·s^−1^ for verapamil with just a single transporter on more than 12% of the occasions (Figure 5), and for 2, 3, 4 and 5 transporters the percentage successes were 23%, 35%, 45% and 54% (the latter marked on the Figure), (iii) for propranolol the success with 5 transporters was 80% and, for a more typical value for *P*_app_ of 10.10^−6^ cm·s^−1^, we could achieve this in 90% of simulations for 5 transporters (Figure 5). (An entirely separate simulation in R – not shown – led to the same conclusion.)

Given that entirely reasonable expectations of transporter expression profiles can thus easily account for the fluxes of even the most rapidly permeable drugs, and even more so for the vast majority of other less permeable drugs, we see no need to invoke bilayer lipoidal permeation at all. In many cases, the transporters involved in Caco-2 transport are entirely well established and leave no room for bilayer lipoidal diffusion. Of course the fact that most drugs have nothing like those large permeabilities means that it is even easier to explain their permeabilities even in terms of ‘random’ expression levels, K_m_ and k_cat_ values (Figure 5).

## Explicability of a Solely Transporter-Mediated Flux of Some Other Drugs

**Fig. 6:**
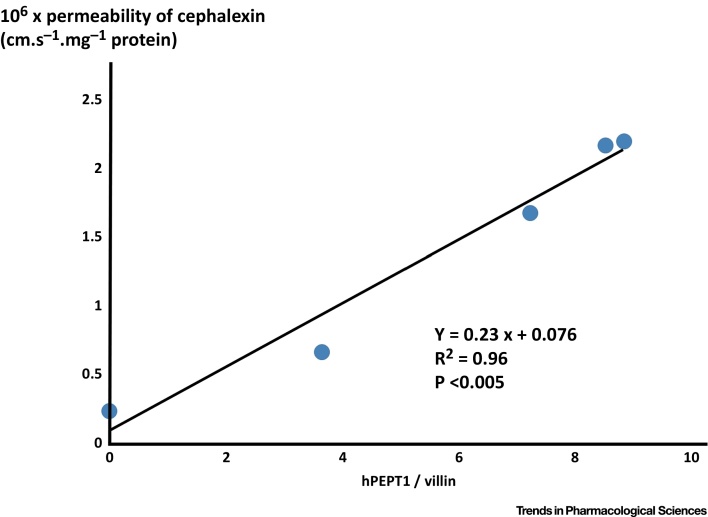
Cephalexin Uptake is Directly Proportional to hPEPT1 Expression. Data are replotted from [66] and show, to a good approximation by varying the transporter expression level, that the ‘background’ uptake rate of cephalexin is negligible.

We noted above the fact [13] that much more than 99% of the transport of sepantronium bromide (YM155) could be shown to pass through a single transporter (SLC35F2), and have stressed [9] that a straightforward way of estimating this is to vary the expression levels of known transporter enzymes. Thus, Chu and colleagues [66] varied the expression level of the PepT1 (SLC15A1) transporter in Caco-2 cells and looked at the effect of this on the transport of cephalexin. We have replotted those data in Figure 6, where it is obvious that, within experimental error, the background rate in the absence of SLC15A1 is indistinguishable from zero. To interpret this, we can do little better than quote the original: “In Caco-2/hPEPT1 cells, an excellent correlation was observed between cephalexin uptake and hPEPT1 expression (R^2^ = 0.96, *P* < 0.005). This demonstrates that cephalexin uptake is directly proportional to hPEPT1 expression” [66].

So, to be clear, even with the most extreme assumptions (most permeable drugs, not recognising all the transporters and their multiple isoforms, no selection for k_cat_, independence from each other of individual transporter expression profiles, k_cat_ and K_m_, etc.) most of the time one can in fact easily account for *P*_app_, simply on the basis of the arguments and data presented [20], for a fully transporter-mediated transport of drugs. There is consequently no need to invoke lipoidal bilayer diffusion at all.

## Two Irrelevancies on which We Have Nothing Discriminating to Say

Matsson *et al*. [20] also make much of two other features: (i) a statement (no actual data are shown) that transport rates are ‘linear’ with substrate concentrations over wide ranges, and that this supposedly cannot be explained by combinations of transporters, and (ii) that equality of transport rates in two directions is hard for transporter-only theories to explain. Regarding (i), we have previously pointed out 6, 9 that, especially in the absence of any knowledge of the transporters involved nor their detailed enzyme kinetics, linearity or its lack is not a criterion of anything (similarly, on the other side, we do not seek to claim that saturation ‘proves’ transporter involvement). Regarding (ii) we have also previously pointed out [6] that, for equilibrative transporters performing facilitated diffusion, this is a simple thermodynamic consequence of the Haldane relation (of enzyme kinetics, that can be read in any suitable textbook such as 67, 68, 69). Specifically, the Haldane relation states that (V_m,f_ × K_m,r_)/(V_m,r_ × K_m,f_) = K_eq_. Not only do transporters explain this bidirectional equivalence of fluxes straightforwardly but it is a necessary fact for enzymes or transporters where K_eq_ = 1. Put another way, for a given external substrate concentration, instantaneous fluxes can differ between the two directions in a Caco-2 set-up even when K_eq_ = 1 (i.e. transport is equilibrative), simply because K_m_ and V_max_ (k_cat_) values can be whatever they are, subject to the constraint of the Haldane relationship. Matsson *et al.* [20] state “equilibrative transporters (which mediate substrate flux along concentration gradients; {their} Box 1) can – under certain circumstances – give rise to direction-independent rates. Thus, near-unity flux ratios do not unambiguously exclude transporter involvement”. Indeed they do not, as when measurements are performed properly (a recent example of near-unity ratios is [70]) they directly reflect the Haldane relationship. Possibly a failure to understand this principle follows from the conflation of two meanings of the word ‘passive’, but we do hope that this particular line of reasoning can be cast properly in the context of the Haldane relationship, which is where it belongs.

## What Criteria Should One Use to Assess the Role of Transporters in Drug Uptake?

We have previously set down why some criteria raised in this debate about the mechanisms of transmembrane drug transport are simply non-discriminatory. We gave two above and others elsewhere [9]. These are not therefore of interest. Much more important is a general strategy used throughout modern molecular genetics to determine the involvement of a gene (product) in a process. This is to vary the expression of the gene product as an independent variable (whether as a knockdown or via a regulatable promoter such as *tet*O [71]), and to observe the effects of that on the dependent process of interest (such as uptake transport). We already gave many hundreds of examples [1]. Similar comments apply to the role of the Henle-Koch postulates in microbiology (e.g. 72, 73).

However, Mattson *et al*. state “At first glance, the transporters only model may appear impossible (or at least extremely daunting) to test: to exhaustively confirm the hypothesis, one would need to identify the missing carriers for all transported drug molecules”. Not at all, and it is no more daunting than seeking the genes (and their products) that are responsible for any biological process of interest. Certainly the first step in any systems biology model is qualitative – to identify the players 7, 8, 18, 19. However, when one has identified them, it is easy to assess their contributions, and we gave examples above (such as that for cephalexin in Figure 6). Indeed Matsson *et al*. [20] later comment “One avenue to identify such novel (sic) drug transporters would be the use of genome-wide single-gene knockout libraries in model organisms like *Saccharomyces cerevisiae*, CRISPR–Cas9 knock-out libraries in human cells, or human haploid genetic screens. Oddly enough this is precisely what we have previously stressed [9], and what we [11] already did (though these papers were not cited by Matsson *et al.* [20]). Others have adopted a similar and highly effective strategy (e.g. [13]) showing extremely clearly that when the pertinent transporters are removed the background uptake (or toxicity of a cytotoxic drug) is negligible. What we now need are QSAR models for each of the main transporter families, to incorporate into the digitally available human metabolic network 8, 74, 75.

## Other Evidence That Protein Carrier-Mediated Transport Is the Dominant Means of Transembrane Uptake of Pharmaceutical Drugs

As we have stressed before (e.g. 6, 9, 10, 76), and we do not repeat the references here, there is considerable evidence for a requirement for transporters for the transmembrane transport of even very small and often hydrophobic molecules. These include alkanes, fatty acids, gases such as CO_2_, O_2_ and NO, ammonia, glycerol and so on, so the bilayer lipoidal permeability in real biological membranes must necessarily be very small. This also provides a ready explanation for a variety of features that are not easily explained (at least without extra *ad hoc* hypotheses) by a view that has it that much or most of the cellular uptake of pharmaceutical drugs occurs through the phospholipid bilayer. Indeed, given that the effect of changing lipids in biophysical terms is not seen as that great, any heterogeneity of uptake between cells, tissues and organisms is most simply explained in terms of the varying expression of the relevant transporters 4, 6, 9, 10. Imaging mass spectrometry (e.g. 77, 78, 79 is beginning to provide outstanding data on the very considerably extent of heterogeneity of drug transport and distribution, while the human proteome atlas [80] and comparable transcriptome data [81] show the equivalent heterogeneity of transporters and other proteins.

## Concluding Remarks

In conclusion (and see also the Outstanding Questions box), the test proposed [20] to see if a random selection from a nominally known distribution of properties of known transporters is a nice idea. Despite the opposite interpretation taken [20], however, the forward modelling data do indeed show that transporters can easily account for the uptake of even the most permeable drugs, even when their permeabilities are given as being several times greater than those of other comparable measurements. This is even more the case for all the other drugs that naturally have considerably lower experimental permeabilities. Parameter estimation data based on selection show it even more clearly. In a similar vein, and famously (if apocryphally^i^), it was suggested that physics-based calculations implied that the bumblebee could not fly. Happily the bumblebees were selected by evolution so that they could, just as transporters were selected to be able to sustain the necessary transport fluxes.

Outstanding Questions What are the quantitative expression profiles of endogenous metabolite transporters (that are also responsible for transporting drugs) between different tissues?

Are these transporters equilibrative or concentrative, and if concentrative what is their mechanism of energy coupling?

What is the detailed enzymology of these transporters, and what are their quantitative structure-activity relationships (QSARs),

How do these vary between different cells, tissues, organisms and species?

How do the uptake profiles between different cells of particular drugs covary with the expression profiles of particular drug transporters, and how might we use these (with the QSARs) to predict the distributions of any drug?

Can we vary the expression profiles (by nutritional, pharmacological or other means) to target specific drugs to specific tissues?

## Note Added in Proof

A recent major review stresses the importance of the issues discussed in [99].

## Figures and Tables

**Figure I:**
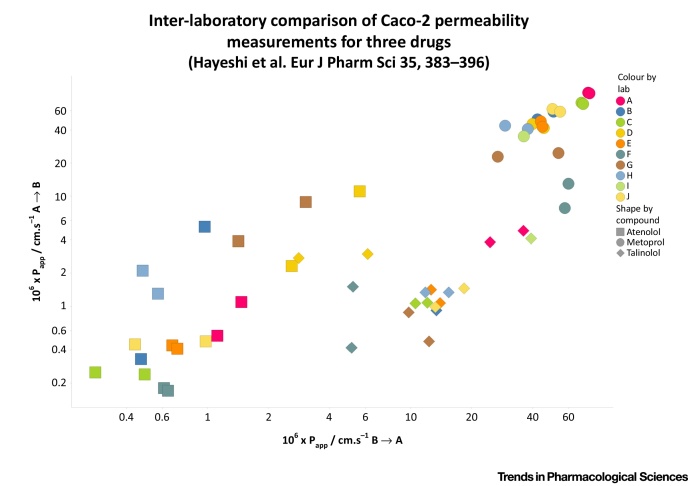
Inter-Laboratory Comparison of Caco-2 Permeability Measurements

**Table 1:** A Comparison of the Values of Caco-2 Permeability Chosen for Verapamil and Propranolol by [20] (and Taken from [29]) with Those Given in Various Other Papers

Compound	10^6^ × Caco-2 *P*_app_ (cm·s^−1^)	Reference
**verapamil**	**1310**	20, 29
	155	[30]
	15.8	[34]
	26.3	[32]
	9.8	[82]
	45.7	[83]
	12.4	[84]
	152	[85]
	62.4	[86]
	69.4	[87]
	22	[88]
	22–24	[89]
	9	[90]
	25	[91]
	22	[92]
**propranolol**	**230**	20, 29
	41.9	[34]
	50	[33]
	27.5	[60]
	29.2	[64]
	25.8	[64]
	44.6	[64]
	39.8	[64]
	57	[64]
	59.7	[64]
	30.1	[61]
	41.7	[32]
	17.5	[82]
	26.3	[63]
	39.8	[83]
	12.9	[84]
	27	[93]
	8–16	[35]
	35.3	[94]
	21.8	[62]
	27.5	[87]
	11.1–27.7	[95]
	16	[88]
	21–36	[89]
	8.2	[96]

## Box 1 Inter-Laboratory Comparison of Caco-2 Permeabilities

Data are replotted from Table 4 of [26] and illustrate that even within labs, and certainly between labs, there can be variations of an order of magnitude or more in Caco-2 permeability measurements. The three drugs shown (atenolol, metoprolol, talinolol) are encoded by shape, and the laboratories by the colour of the symbols. There were two separate ‘batches’ of Caco-2 cells tested (Figure I).

## Box 2 Materials and Methods and Relevant Calculations

As described in the supplementary material of Matsson et al. [20], an apparent permeability, *P*_app_, can be calculated from the flux of a drug passing through one or several transporters. First the steady state flux, *J*_*i*_, at which the drug passes through each transporter is calculated using the Henri-Michaelis-Menten equation, given: the concentration of the drug ([*D*]), the concentration of the transporter ([*T*_*i*_]_0_), the area (*A*), the area density of proteins in the membrane (*α* = 0.5 mg·cm^−2^), the turnover number *k*_*cat,i*_, and the Michaelis constant *K*_*m,i*_. The sum of the steady-state fluxes (*J*_*tot*_) through all the transporters is then the total steady-state flux of drug entry:

(1) $$J_{tot}=\sum_{i}J_{i}=\sum_{i}\frac{{\left[T_{i}\right]}_{0}\times A\times \alpha \times k_{cat,i}\times [D]}{K_{m,i}+[D]}$$

That steady-state flux of drug entry would correspond to a certain apparent permeability (*P*_app_) through the following equation:

(2) $$P_{app}=\frac{J_{tot}}{A\times \frac{\left[D\right]}{1000}}=\frac{J_{tot}\times 1000}{A\times [D]}\text{,}$$

where *A* is the area of the membrane in the permeability assay, taken as 0.33 cm^2^ (Matsson, personal communication). The factor 1000 converts the concentration of the drug ([D]) from pmol·L^−1^ to pmol·cm^−3^. We constructed a kinetic model in the software COPASI [97] version 4.15^ii^ that incorporates Eqs (1) and (2) and supply this model as supplementary data in the SBML format [98]. We then used this model to a) find many sets of parameter values that lead to rates of entry through a single transporter equivalent to the permeability of verapamil, and b) generate 10,000 models with those parameters sampled randomly from appropriate distributions (see supplementary data) with 1, 2 and 5 transporters. We provide COPASI native files for both a) and b) in the supplement.

It is, as usual, necessary that all numbers entered in Eqs (1) and (2) be in compatible (i.e. self-consistent) units. Thus to make this process more transparent, we converted all data in the supplementary material of Matsson et al. [20] to compatible units as follows:

• transporter concentrations ([*T*_*i*_]_0_): pmol·mg^−1^ total protein;
• drug concentrations ([D]: pmol·L^−1^
• area (*A*): cm^2^
• protein area density: mg·cm^−2^
• turnover numbers (*k*_*cat,i*_): s^−1^
• Michaelis constant (*K*_*m,i*_): pmol·L^−1^
• fluxes (*J*_*tot*_ and *J*_*i*_): pmol·s^−1^
• apparent permeability (*P*_*app*_): cm·s^−1^

## Resources

^i^ http://en.wikipedia.org/wiki/Bumblebee#Misconception_about_flight

^ii^ www.copasi.org
