# Supplementary material for: Fitting Transporter Activities to Cellular Drug Concentrations and Fluxes: Why the Bumblebee Can Fly
Source: Trends Pharmacol Sci. 2015 Nov;36(11):710–23. doi: 10.1016/j.tips.2015.07.006 (PMC4642801; doi:10.1016/j.tips.2015.07.006)
Supplement: Supplementary file 1 [file mmc1.pdf]

Fitting standard distributions to data of Matsson *et al.*

Pedro Mendes, Stephen G. Oliver, Douglas B. Kell

17-06-2015

1. Introduction

Matsson *et al.* (2015) provide data for transporter concentration (hereafter "*T0*") and k_{cat}, and histograms of K_m. They sampled values from the empirical distributions of their data, however in our software COPASI this is not possible; rather values must be drawn from normal, log-normal or gamma distributions. Thus we need to fit their data to the best of those three distributuions. Visually it is clear that the data are not normal, and thus here we fit them to log-normal and gamma distributions. Fits are carried out through maximum likelyhood estimation and selection of best fit was carried out based on Akaike's information criterion.

Estimation of parametric distribution coefficients was carried out with R (R Core Team, 2014) using the `fitdistrplus` package (Pouillot & Delignette-Muller, 2010). The code is reproduced in this document. The best fit distributions were then used with the modelling software COPASI (Hoops *et al.*, 2006) to generate 10,000 different models (of a single transporter) by random sampling from those distributions. The COPASI sampled parameters are compared here to the original data and the density of the distributions.

2. Data

K_m

K_m data is, unfortunately, given as a frequency histogram, but we need a full set of data to fit to distributions. A surrogate data set is then created by repeating the midpoint in each bin as many times as the frequency of that bin. The new data set is then ready for the MLE fit.

```r
# the (given) break points for the bins for Km histogram
kmbins = c(1e-3, 10^(-2.5), 1e-2, 10^(-1.5), 1e-1, 10^(-0.5), 1, 10^(0.5), 10,
           10^(1.5), 1e2, 10^(2.5), 1e3, 10^(3.5), 1e4, 10^(4.5), 1e5)
# the (calculated) midpoints of the histogram bins
kmmids = c( 10^(-2.75), 10^(-2.25), 10^(-1.75), 10^(-1.25), 10^(-0.75), 10^(-0.25),
            10^(0.25), 10^(0.75), 10^(1.25), 10^(1.75), 10^(2.25), 10^(2.75),
            10^(3.25), 10^(3.75), 10^(4.25), 10^(4.75) )
# frequency for SLC and ABC transporters, given the break points above
FreqSLC = c(1,3,2,15,15,21,37,73,102,60,47,28,22,15,3,1)
FreqABC = c(0,0,1,10,11,19,29,62,56,32,34,23,11,5,0,0)
# because we only have histogram, we have to create a surrogate data series
# according to histogram (repeating the midpoint Freq number of times)
km <- rep(kmmids,FreqSLC)
```

T0

T0 data (transporter concentration) is given explicitly in fmol/ mg total protein. However we need to convert this to pmol/mg total protein (a factor of 10^{-3}).

```
t0 <- c(34200, 12200, 10600, 10500, 9000, 7700, 7010, 5880, 4870, 3370, 2730,
        2710, 2450, 2320, 2320, 2170, 2160, 2030, 1750, 1710, 1690, 1630, 1540,
        1440, 1300, 1280, 1280, 1230, 1210, 1090, 1020, 980, 980, 790, 780,
        650, 620, 590, 550, 490, 470, 470, 470, 460, 440, 380, 360, 300, 290,
        290, 280, 260, 240, 210, 210, 200, 200, 180, 180, 150, 150, 140, 120,
        120, 120, 110, 110, 110, 99, 97, 89, 85, 84, 79, 77, 75, 73, 72, 66,
        62, 62, 57, 52, 46, 44, 43, 43, 42, 37, 36, 35, 32, 30, 27, 26, 24, 22,
        17, 13, 12)
t0 <- t0 * 1e-3
```

k_{cat}

k_{cat} data is also given explicitly:

```
kcat <- c( 14, 10, 9.2, 6, 5.8, 3, 1, 5, 2, 1200, 450, 966, 567, 280, 1000,
          650, 600, 57, 30, 25, 11, 20, 13, 6, 2.2, 1.2, 3.7, 2.8, 0.6, 1, 1,
          1, 0.7, 0.3, 130, 70, 85, 4, 5, 1.5, 0.2, 2.5, 0.5, 0.12, 69, 8,
          300)
```

3. Maximum likelyhood estimation of distribution parameters

K_m

```
library(fitdistrplus)
kmfitG <- fitdist(km,"gamma",start=list(scale=1,shape=1), lower=1e-8)
kmfitLN <- fitdist(km,"lnorm")
# compare goodness of fit estimates
summary(kmfitLN)
```

```
## Fitting of the distribution ' lnorm ' by maximum likelihood
## Parameters :
##         estimate Std. Error
## meanlog 2.981718 0.13414454
## sdlog   2.829782 0.09485446
## Loglikelihood:  -2421.181   AIC:  4846.362   BIC:  4854.559
## Correlation matrix:
##         meanlog sdlog
## meanlog       1     0
## sdlog         0     1
```

```
summary(kmfitG)
```

```
## Fitting of the distribution ' gamma ' by maximum likelihood
## Parameters :
##           estimate   Std. Error
## scale 2757.8722975 316.74770265
## shape    0.2141684   0.01106001
## Loglikelihood:  -2539.008   AIC:  5082.015   BIC:  5090.211
```

```
## Correlation matrix:
##            scale      shape
## scale  1.0000000 -0.4505311
## shape -0.4505311  1.0000000
```

```r
gofstat(list(kmfitLN,kmfitG), fitnames=c("lnorm","gamma"))
```

```
## Goodness-of-fit statistics
##                                  lnorm      gamma
## Kolmogorov-Smirnov statistic 0.1190807  0.2659812
## Cramer-von Mises statistic   0.9712746  7.2098402
## Anderson-Darling statistic   4.8653835 34.8105455
##
## Goodness-of-fit criteria
##                                  lnorm    gamma
## Aikake's Information Criterion 4846.362 5082.015
## Bayesian Information Criterion 4854.559 5090.211
```

```r
cdfcomp(list(kmfitLN,kmfitG), legendtext=c("lnorm","gamma"),xlogscale=T, main="Km")
```

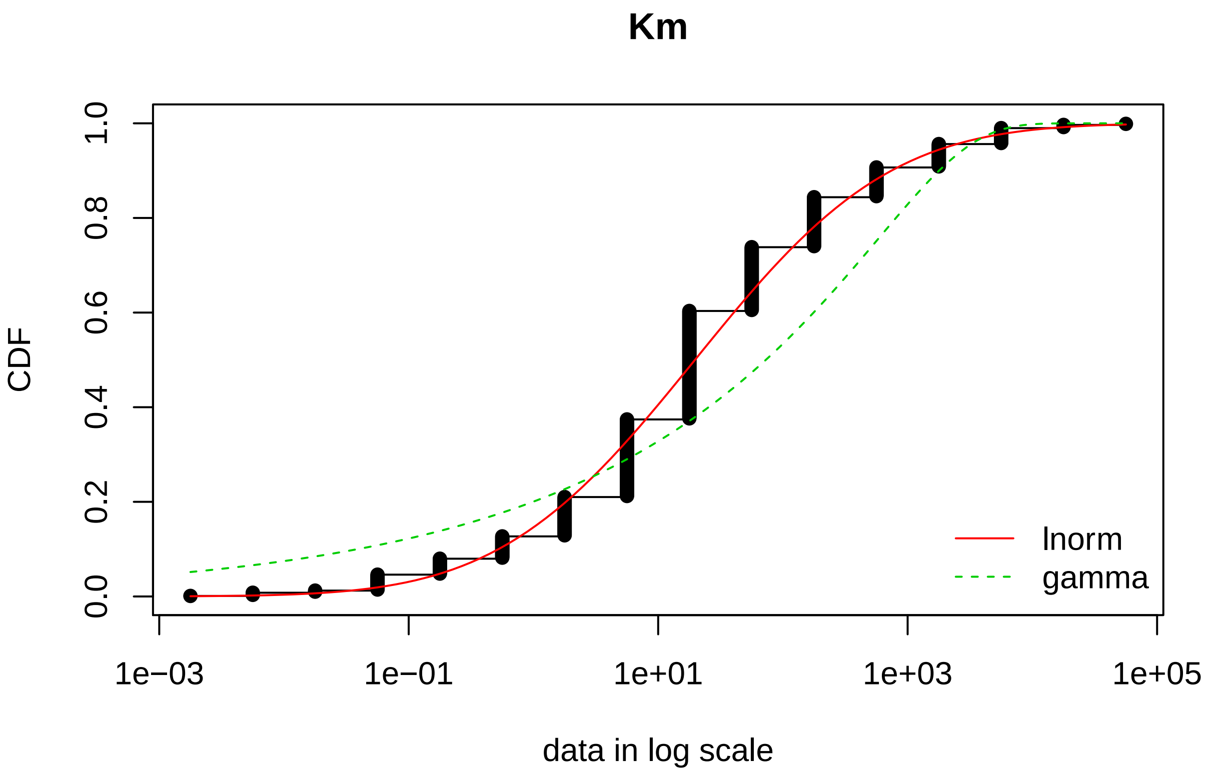

The log-normal distribution with meanlog=2.981718 and sdlog=2.829782 is adopted to describe the K_m data as it has a lower AIC value and the distribution is clearly closer to the (surrogate) data.

T0

```r
t0fitG <- fitdist(t0,"gamma",start=list(scale=1,shape=1), lower=1e-8)
t0fitLN <- fitdist(t0,"lnorm")
# compare goodness of fit estimates
summary(t0fitLN)
```

```
## Fitting of the distribution ' lnorm ' by maximum likelihood
## Parameters :
##          estimate Std. Error
## meanlog -1.121480  0.1793716
## sdlog    1.793716  0.1268347
## Loglikelihood:  -88.17483   AIC:  180.3497   BIC:  185.56
## Correlation matrix:
##               meanlog         sdlog
## meanlog  1.000000e+00 -4.041308e-11
## sdlog   -4.041308e-11  1.000000e+00
```

```r
summary(t0fitG)
```

```
## Fitting of the distribution ' gamma ' by maximum likelihood
## Parameters :
##        estimate Std. Error
## scale 3.7939727 0.73237923
## shape 0.4151268 0.04764835
## Loglikelihood:  -107.1486   AIC:  218.2972   BIC:  223.5075
## Correlation matrix:
##            scale      shape
## scale  1.0000000 -0.5946002
## shape -0.5946002  1.0000000
```

```r
gofstat(list(t0fitLN,t0fitG), fitnames=c("lnorm","gamma"))
```

```
## Goodness-of-fit statistics
##                                   lnorm     gamma
## Kolmogorov-Smirnov statistic 0.09117613 0.1455117
## Cramer-von Mises statistic   0.12856200 0.7401482
## Anderson-Darling statistic   0.76992474 4.1049645
##
## Goodness-of-fit criteria
##                                   lnorm    gamma
## Aikake's Information Criterion 180.3497 218.2972
## Bayesian Information Criterion 185.5600 223.5075
```

```r
cdfcomp(list(t0fitLN,t0fitG), legendtext=c("lnorm","gamma"),xlogscale=T, main="T0")
```

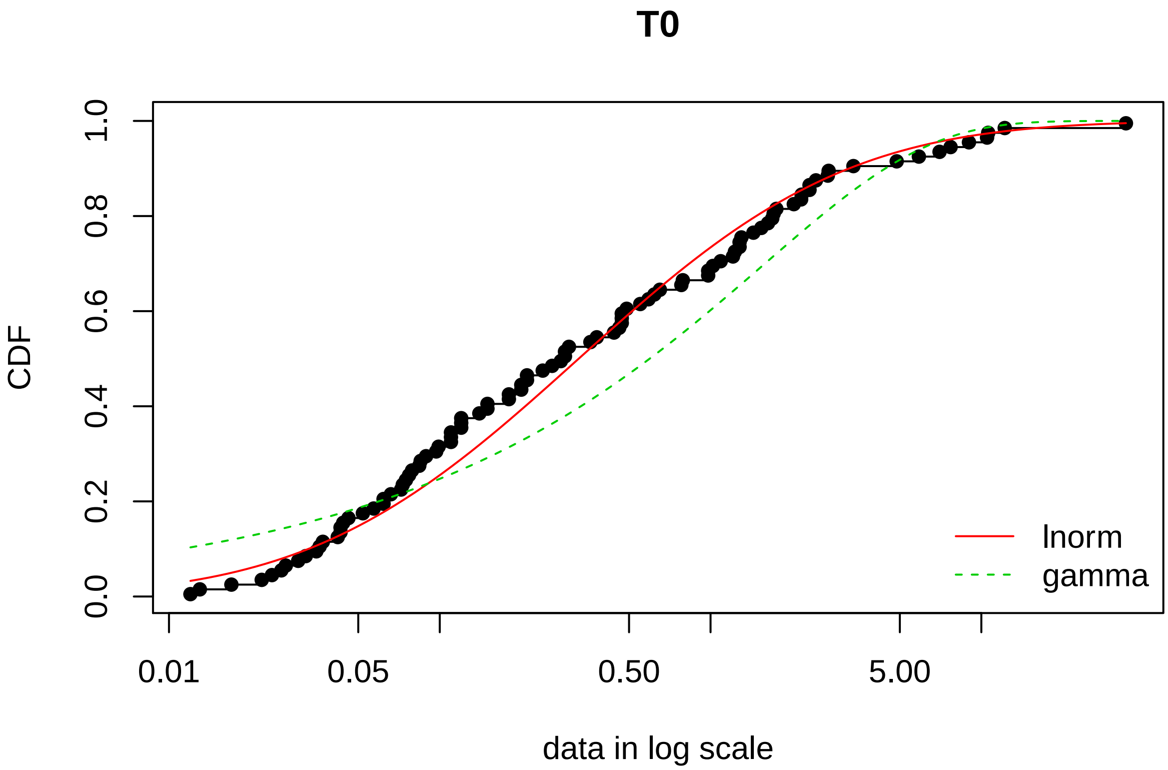

The log-normal distribution with meanlog=-1.121480 and sdlog=1.793716 is adopted to describe the *T0* data as it has a lower AIC value and the distribution is clearly closer to the (surrogate) data.

k_{cat}

```
kcatfitG <- fitdist(kcat,"gamma",start=list(scale=1,shape=1), lower=1e-8)
kcatfitLN <- fitdist(kcat,"lnorm")
# compare goodness of fit estimates
summary(kcatfitLN)
```

```
## Fitting of the distribution ' lnorm ' by maximum likelihood
## Parameters :
##         estimate Std. Error
## meanlog 2.405581  0.3626783
## sdlog   2.486397  0.2564521
## Loglikelihood:  -222.5617   AIC:  449.1233   BIC:  452.8236
## Correlation matrix:
##               meanlog         sdlog
## meanlog  1.000000e+00 -6.608731e-10
## sdlog   -6.608731e-10  1.000000e+00
```

```
summary(kcatfitG)
```

```
## Fitting of the distribution ' gamma ' by maximum likelihood
## Parameters :
##          estimate   Std. Error
```

```
## scale 511.8383572 164.59911965
## shape   0.2751321   0.04450247
## Loglikelihood:  -231.3404   AIC:  466.6809   BIC:  470.3812
## Correlation matrix:
##            scale      shape
## scale  1.0000000 -0.5026713
## shape -0.5026713  1.0000000
```

```r
gofstat(list(kcatfitLN,kcatfitG), fitnames=c("lnorm","gamma"))
```

```
## Goodness-of-fit statistics
##                                  lnorm     gamma
## Kolmogorov-Smirnov statistic 0.1081347 0.2286459
## Cramer-von Mises statistic   0.1185118 0.5989112
## Anderson-Darling statistic   0.7649438 2.9766586
##
## Goodness-of-fit criteria
##                                  lnorm    gamma
## Aikake's Information Criterion 449.1233 466.6809
## Bayesian Information Criterion 452.8236 470.3812
```

```r
cdfcomp(list(kcatfitLN,kcatfitG), legendtext=c("lnorm","gamma"),xlogscale=T, main="kcat")
```

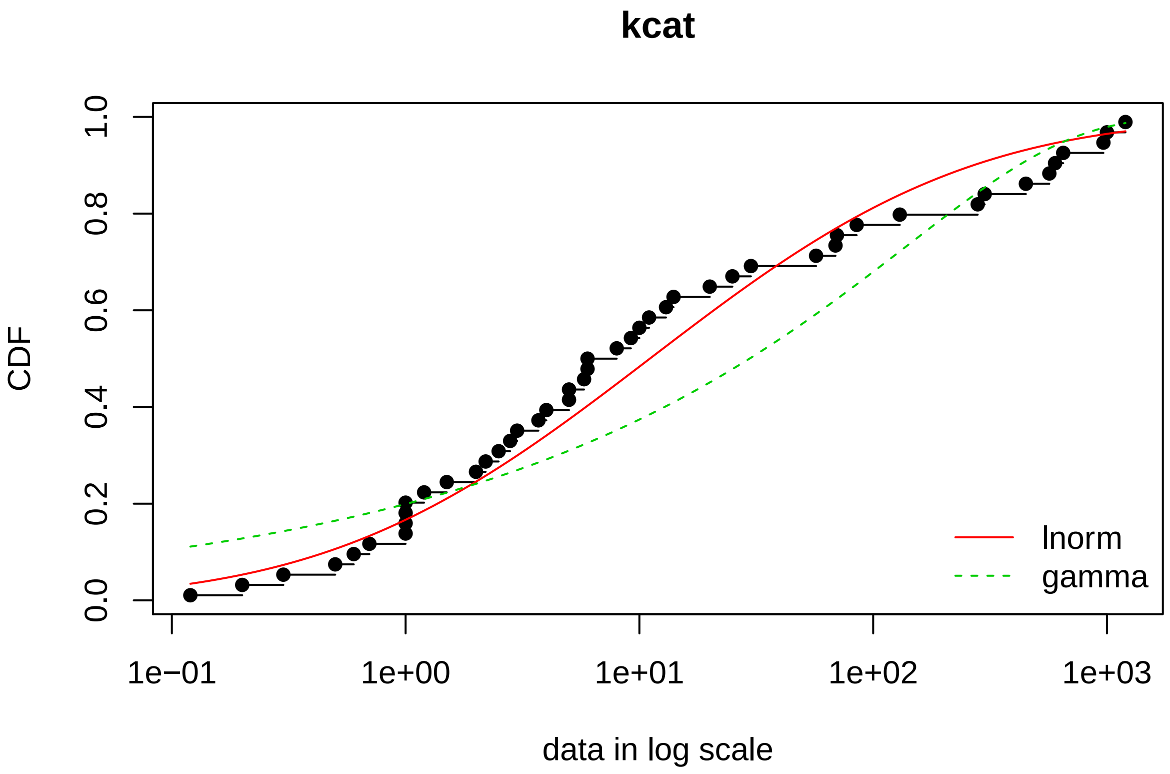

The log-normal distribution with meanlog=2.405581 and sdlog=2.486397 is adopted to describe the k_{cat} data as it has a lower AIC value and the distribution is clearly closer to the (surrogate) data.

Simulated data *versus* given data

The log-normal distributions obtained for each of the parameters were then used in COPASI to sample 10,000 combinations of random values for the three parameters. We now compare the data simulated with COPASI to the original data in order to visually inspect the extent of agreement between the empirical and the MLE log-normal distributions.

```
# read the sampling data simulated with COPASI
myData = read.table("sampling10k.txt")
```

K_m

```
library(ggplot2)
# density functions for plotting fitted distributions
kmg = rlnorm(1000000, 2.981718, 2.829782)
ggplot() +
  geom_histogram(aes(x=km,y=..density..), binwidth=0.5, fill="red", alpha=0.25) +
  scale_x_log10() + theme_bw() +
  geom_histogram(aes(x=myData[[1]], y=..density..), binwidth=0.5, fill="blue", alpha=0.25) +
  geom_density(aes(x=kmg, y=..density..), line="black")
```

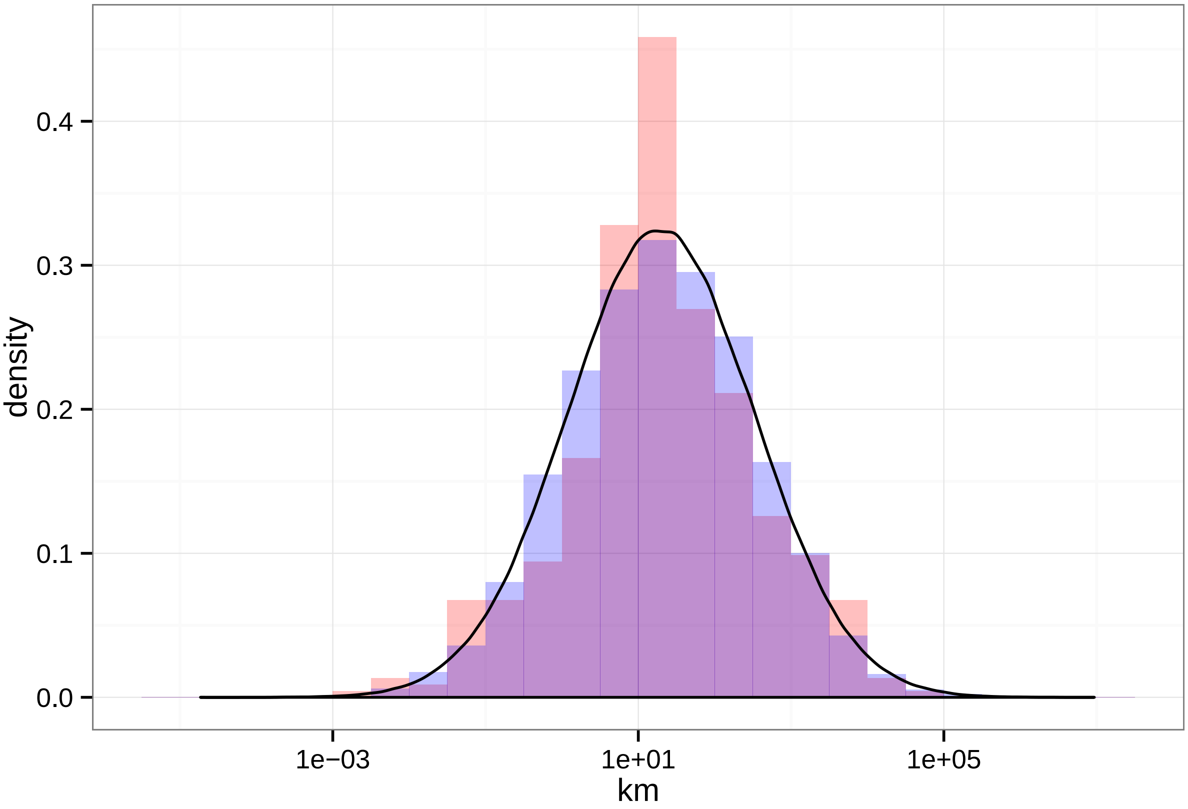

Histogram in red corresponds to the data in Matsson *et al.* (2015), histogram in blue to the data generated with COPASI. Black line is the density of the log-normal distribution with the fitted parameters.

T0

```
t0g = rlnorm(1000000, -1.121480, 1.793716)
ggplot() +
  geom_histogram(aes(x=t0,y=..density..), binwidth=0.1, fill="red", alpha=0.25) +
  scale_x_log10() + theme_bw() +
  geom_histogram(aes(x=myData[[3]], y=..density..), binwidth=0.1, fill="blue", alpha=0.25) +
  geom_density(aes(x=t0g, y=..density..), line="black")
```

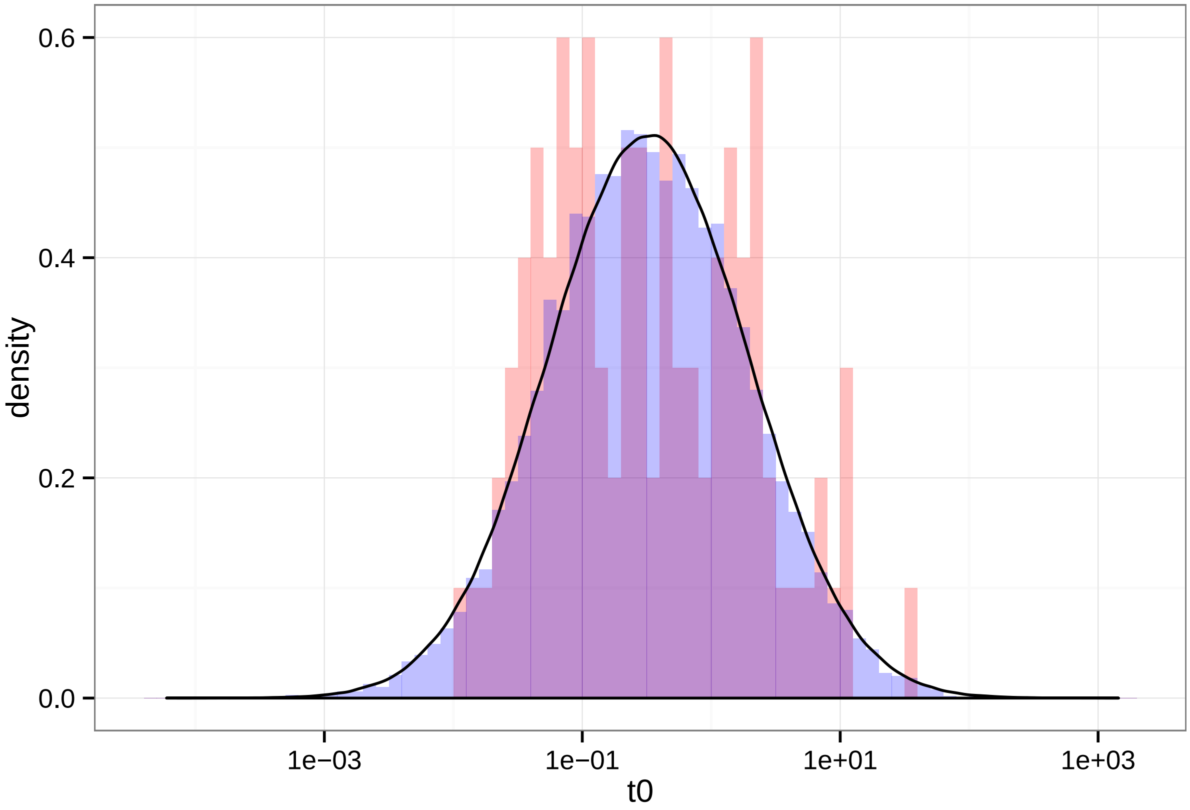

Histogram in red corresponds to the data in Matsson *et al.* (2015), histogram in blue to the data generated with COPASI. Black line is the density of the log-normal distribution with the fitted parameters.

k_{cat}

```
kcatg = rlnorm(1000000, 2.405581, 2.486397)
ggplot() +
  geom_histogram(aes(x=kcat,y=..density..), binwidth=0.25, fill="red", alpha=0.25) +
  scale_x_log10() + theme_bw() +
  geom_histogram(aes(x=myData[[2]], y=..density..), binwidth=0.25, fill="blue", alpha=0.25) +
  geom_density(aes(x=kcatg, y=..density..), line="black")
```

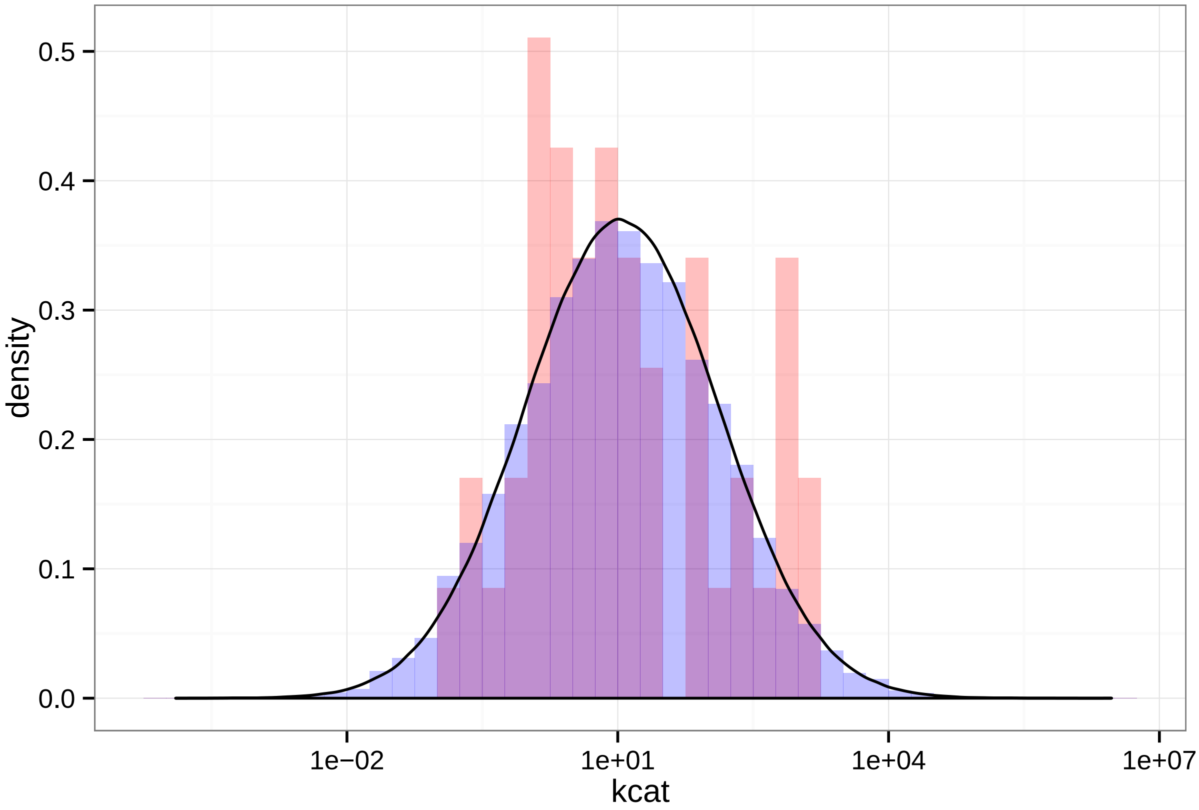

Histogram in red corresponds to the data in Matsson *et al.* (2015), histogram in blue to the data generated with COPASI. Black line is the density of the log-normal distribution with the fitted parameters.

4. Conclusion

The log-normal distributions are sufficiently close to the empirical distributions and therefore were adopted for this study. They were used to estimate the proportion of times that systems with 1, 2, or 5 transporters are able to match the permeability of verapamil (1310 x 10^{-6} cm/s).

5. References

Hoops S., Sahle S., Gauges R., Lee C., Pahle J., Simus N., Singhal M., Xu L., Mendes P. & Kummer U. (2006) COPASI — a COmplex PAthway SImulator. *Bioinformatics* **22**, 3067-74.

Matsson P, Fenu L.A., Lundquist P., Wiśniewski J.R., Kansy M. & Artursson P. (2015) Addendum to 'Quantifying the impact of transporters on cellular drug permeability', *Trends in Pharmacological Sciences*.

Pouillot R. & Delignette-Muller M.L. (2010) Evaluating variability and uncertainty separately in microbial quantitative risk assessment using two R packages, *International Journal of Food Microbiology* **142**, 330-40.

R Core Team (2014) R: A Language and Environment for Statistical Computing, *R Foundation for Statistical Computing*: Vienna, Austria. http://www.R-project.org
